# Effort expectation and strategic cue use in visual search

**DOI:** 10.3758/s13415-025-01358-1

**Published:** 2025-11-11

**Authors:** Sizhu Han, Jan Tünnermann, Sara Katharina Hornung, Anna Schubö

**Affiliations:** Cognitive Neuroscience of Perception and Action, Department of Psychology, Philipps-Universität Marburg, Gutenbergstraße 18, 35032 Marburg, Germany

**Keywords:** Search difficulty, Effort expectation, Cue-guided attention

## Abstract

Positive cues provide information about target features, facilitating processing of the upcoming input. The role of “negative cues,” which signal distractor features, is less clear. Their use seems to incur a certain mental effort, and consequently, their effectiveness could depend on whether the benefit of reducing search effort outweighs the effort required to use them. Crucially, when search effort is predictable, participants can rely on expected effort in deciding whether to use a negative cue. This subjective effort/benefit evaluation may explain the inconsistent effects sometimes observed with negative cues. However, the influence of expected effort has not yet been examined in this context. Varying effort expectation may provide a way to test this prediction. To this end, we manipulated effort expectation by embedding a small number of easy trials within blocks of mainly difficult trials and vice versa. We assumed that expected effort can boost cue utilization, especially when a difficult search was expected. The impact of positive and negative cues was compared to neutral cues under identical expectations. We found that benefits of positive cues increased when search was more effortful. Negative cueing effects, however, showed no reliable benefits, even when higher effort was expected. Interestingly, search performance was better when the expected effort matched actual task difficulty, though this pattern was more robust in difficult compared to easy trials. This asymmetry reveals an interaction between effort expectation and task difficulty. We discuss the neural underpinnings of how these factors might influence visual search.

**Supplementary Information:**

The online version contains supplementary material available at 10.3758/s13415-025-01358-1.

## Introduction

In daily life, we routinely engage in visual searches, such as locating keys in a bag, a peeler in the cutlery drawer, or apples on a supermarket shelf. These tasks rely on our attention system, which prioritizes goal-relevant targets while avoiding distractors that are not relevant for the ongoing task (Desimone & Duncan, 1995; Wolfe & Horowitz, 2017). Notably, this process is not always effortless, depending on the difficulty of the search (Anderson & Lee, 2023). This difficulty is often quantified by “search slopes,” a measure of how search time increases with the set size of items on a display (Bacon & Egeth, 1994). However, there are also other factors that determine difficulty as evidenced by their impact on the slope, such as the similarity between targets and distractors (Duncan & Humphreys, 1989). When their similarity is high, search can be cognitively demanding, requiring effort to resolve ambiguity.

One mechanism which organisms employ to mitigate effortful search is integrating information about the target that is available before the search. Consider searching for red apples in a supermarket. Searchers often rely on a target template, a representation of target features, to prioritize the processing of objects that match these features. For instance, the apple’s red color and its roundish shape could be included in the template. Decades of laboratory research have collected robust evidence for this mechanism: when participants of search experiments are given a positive cue in advance that informs about a target feature, their attention can be guided more efficiently to the target, speeding up response times and improving report accuracy (Wolfe, 2020, 2021). However, the role of “negative cues” – foreknowledge of a distractor feature (e.g., avoid green apples) – remains contentious. While some studies have reported faster target detection with negative cues (Arita et al., 2012; Conci et al., 2019; Zhang & Carlisle, 2023; Zhang et al., 2022), others found no benefits or even slower responses compared to non-informative cues (Beck & Hollingworth, 2015; Becker et al., 2015; Berggren & Eimer, 2021; Salahub & Emrich, 2021). These mixed results are at odds with the wealth of findings on positive cues that seem to be universally effective. Even when negative cues were found to be helpful, their benefits were smaller than those of positive cues (Arita et al., 2012; Conci et al., 2019; Zhang & Carlisle, 2023; Zhang et al., 2022). Some studies documented that participants simply ignore negative cues when alternative search strategies exist that are less demanding than utilizing negative cues (Kerzel & Huynh Cong, 2022; Rajsic et al., 2020). For instance, when provided with positive and negative cues simultaneously, participants used only positive cues to find the target (Rajsic et al., 2020). A possible reason for the elusive negative cueing effect might be that, compared to positive cues, utilizing negative cues comes with a higher mental effort which searchers try to avoid, unless the reward of using the cue (e.g., finding the target more quickly) outweighs the effort in setting it up.

A trade-off between cue use effort and search effort seems also likely when considering studies that varied the search difficulty. For instance, Conci and colleagues (2019) asked participants to identify a T-shaped target among seven L-shaped distractors, with half of the items in one color and the other half in another. Prior to search display onset, a cue indicated either the target color (a positive cue), the distractor color (a negative cue), or a neutral color. The difficulty between participant groups was varied by making distractors more or less similar to the target. For the easy search group, negative cues offered no advantage over neutral cues, whereas in the difficult search group, participants benefited from negative cues. Following Conci et al.’s (2019) design, Zhang and colleagues (2022) tested whether the differential negative cueing effects depended on earlier experience of the difficulty levels and conducted a within-block design where easy and difficult trials switched at the middle of blocks, with the order counterbalanced across participants. Their results again showed higher negative cueing effects with the more difficult task, and no carry-over effects of ignoring the negative cue from earlier experience in easier search. This indicates that participants can flexibly adjust attentional strategies to the task at hand (Zhang et al., 2022). Negative cueing effects were also reported by Arita et al. (2012), who found them to be stronger with larger set sizes. Taken together, these findings suggest that participants are more willing to use negative cues when search is more effortful. On a mechanistic level, confronted with sufficiently effortful searches, participants might use negative cues to actively suppress distractors to aid finding the target (see also Carlisle, 2023; Zhang & Carlisle, 2023), or to rapidly disengage attention from distractors (Geng, 2014; Moher & Egeth, 2012; Zhang et al., 2022).

Critically, what constitutes “effortful” may not be the same for all individuals. The subjective evaluation of effort may explain the high individual differences observed in negative cueing effects, even if search difficulty remains the same throughout the experiment (Chidharom & Carlisle, 2024; Heuer & Schubö, 2020). One recent study revealed that participants with higher proactive control efficiency are more likely to use negative cues to aid search (Chidharom & Carlisle, 2024). Following the reasoning above, the proactive control advantage could reduce the effort of preparing distractor templates, making their employment more likely.

A trade-off between cue use effort and the reward it affords – may it be faster search or reduced experienced search effort – also seems plausible in light of studies examining other effort types involved in visual search. Research on adaptive-choice visual search – where participants can choose between two targets – shows that individuals adjust their attentional control settings in response to dynamically changing distractor contexts. However, participants seem to delay these updates, possibly due to the effort involved in switching search templates (Bergmann et al., 2020; Irons & Leber, 2016; Mu et al., 2024). That is, when confronted with potential target sets of different size, participants tend to search through slightly larger sets than necessary, postponing a template switch until the effort of continued inefficient search outweighs the effort of switching. Similarly, in visual foraging tasks where participants collect multiple items, they tend to avoid switching between target types, especially when searching for difficult conjunction targets (e.g., Kristjánsson et al., 2014) or when highly specific target feature templates are required – likely due to the high mental effort involved in fine-tuning the existing template with respect to the current context (Tünnermann et al., 2021). Instead, foragers seem to invest more search effort in locating targets at larger distances apart (and the physical effort for making larger movements) to continue with the existing template and avoid the costly template switches. In experiments where searchers choose between sets that differ in set size and target discriminability, they balance these factors to optimize performance and potentially minimize overall effort (Anderson, 2021) – for example, opting to search through a larger set when the target (such as a Landolt C with a large opening) is easier to identify (Henare et al., 2024; Wagner et al., 2023, 2025). In sum, participants seem to endure additional search effort until the effort of switching or setting up a new template is justified by the perceived reward.

Although we consider the proposed role of search effort trade-offs in negative cue use to be highly plausible, this idea currently lacks direct empirical support. One challenge is the difficulty of measuring how observers subjectively gauge mental effort. The actual effort in a search task is only experienced after the onset of the search display, once the cue has already disappeared. However, much of the effort required to implement cue use likely needs to take place beforehand, while the cue is still present. Therefore, if the visual system takes search effort into account when deciding whether to use a negative cue, it must rely on *expected effort*, with these expectations being shaped by prior experience. In earlier studies on negative cue use (e.g., Conci et al., 2019; Zhang et al., 2022), participants could predict upcoming search effort, equating expected and actual search effort, because difficulty either remained the same throughout the experiment or remained predictable within blocks. The present study is designed to disentangle actually experienced effort from expected effort.

To modulate effort expectation, we embedded a small number of easy trials within blocks of mostly difficult trials, or vice versa. Task difficulty was manipulated by making the target’s orientation more or less similar to the distractor’s orientation. Consequently, participants could expect difficult searches in blocks with a large proportion of difficult trials and easy searches in blocks with predominantly easy trials. Importantly, some trials violated these expectations, such as easy trials occurring within blocks largely composed of difficult trials. We provided a color cue before each search display to accurately inform participants about the color of the upcoming target (i.e., a positive cue) or distractors (i.e., a negative cue). A neutral color that would not appear in subsequent search served as a baseline. Comparison with this condition allowed us to measure the effectiveness of the negative or the positive cue. If effort expectation boosts the utility of negative cues when expecting a difficult search, participants may be more willing to use negative cues. In this case, negative cueing effects might be more likely to occur or perhaps enlarged compared to expected easy trials. We analyzed the data with a repeated-measures ANOVA as commonly done in this type of study. We further employed Bayesian multi-level modelling for a more fine-grained analysis, as potentially small negative cueing effects and their interaction with effort expectation could be hidden due to the averaging or the imbalanced cell population not accounted for in the ANOVA.

To foreshadow our findings, the negative cueing effects remained elusive once more; interestingly, the manipulation of expected effort seemed to interact with positive cueing effects. We discuss the boundary conditions of negative cue use, the role of effort expectation in positive cue use and its neural underpinnings.

## Methods

### Participants

Thirty participants completed the experiment (18 female, 12 male; age: M = 23.93; SD = 2.75 years). One participant failed the practice and was replaced with a new participant. A minimum sample size of 24 was determined based on a priori power analysis (using G*Power 3.1, Faul et al., 2007), with an assumed power of.80 at an alpha level of.05, for moderate effect sizes (d of.60). This setup was informed by earlier studies that reported RT benefits with negative cues (Arita et al., 2012; Conci et al., 2019; Zhang & Carlisle, 2023; Zhang et al., 2022)⁠.

All participants had normal or corrected-to-normal vision. Visual acuity and color vision were tested with the OCULUS Binoptometer 3 (OCULUS Optigkeräte GmbH, Wetzlar, Germany). All participants provided written informed consent prior to the experiment and were compensated with course credits or 10 € per hour. The experimental procedure was approved by the ethics commission of the Department of Psychology, Philipps-Universität Marburg, and conducted in accordance with the 1964 Declaration of Helsinki.

### Apparatus and stimuli

The experiment took place in a dimly lit, sound-attenuated room. Participants were seated on a comfortable chair at a distance of 100 cm to a 22-in. VPixx VIEWPixx monitor (Vpixx Technologies Inc.). The experiment was run with E-Prime 2.0 (Psychology Software Tools, Sharpsburg, PA) on a Windows 7 computer (1,680 × 1,050 pixels, screen resolution at 60 Hz refresh rate).

All stimuli were presented on a black background (RGB [0, 0, 0], 0.28 cd/m^2^). Gabor patches (with a radius of 0.81° visual angle, cosine envelope, frequency of 0.05 and deviation of 12 pixels) were generated via the online Gabor patch generator (https://www.cogsci.nl/gabor-generator). They were tilted to one of six orientations (0°, 90°, ± 15° and ± 45° relative to vertical midline) and displayed in one of four isoluminant colors: yellow (RGB [167, 93, 0], 25.30 cd/m^2^); green (RGB [0, 142, 0], 25.60 cd/m^2^); blue (RGB [0, 111, 234], 25.61 cd/m^2^); red (RGB [255, 0, 0], 25.20 cd/m^2^).

**Fig. 1:**
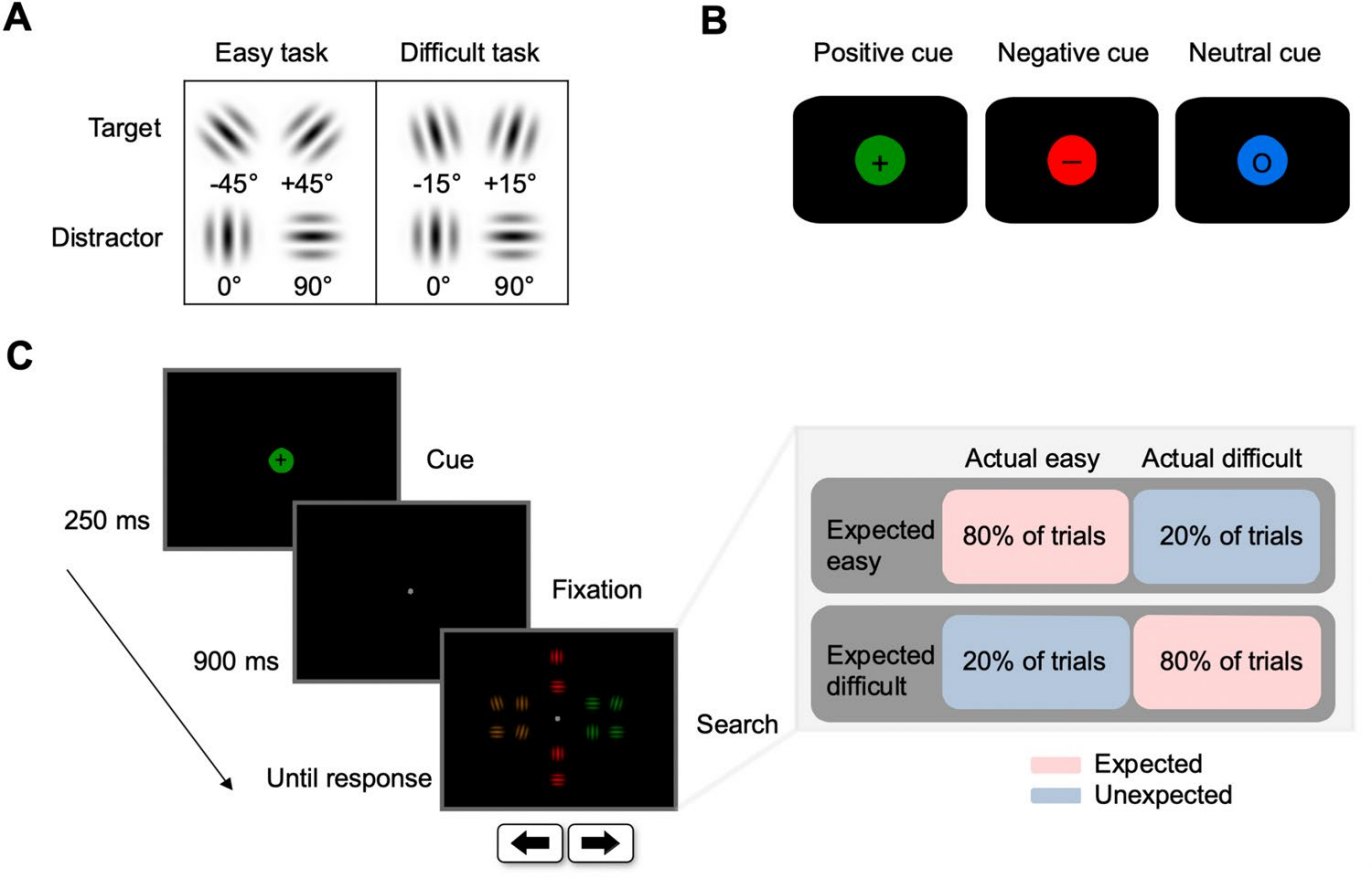
Experimental design. (**A**) Orientations of the target and distractors in the easy and difficult search task. (**B**) A “ + ” symbol at the center of a colored circle indicated a positive cue, signaling the color of the upcoming target; a “ − ” symbol indicated a negative cue, signaling the color of the upcoming distractors; an “○” symbol indicated a neutral cue, signaling a color that did not appear in the upcoming search display. The denoted color is an example, and each cue can be any of the three colors. (**C**) Trial sequence (left) and effort expectation manipulation (right). Different cue types were presented in separate blocks. In blocks where participants expected an easy search task, 20% difficult trials were randomly embedded in 80% of trials with the easy task. In blocks where participants expected a difficult search task, 20% easy task trials were randomly embedded in 80% of trials with the difficult task. Note that colored items were always spatially grouped by color, consistent with the given example, but color-location bindings were randomized across trials. Stimulus sizes are not representative

Each search array consisted of four yellow Gabor patches and eight patches of two other colors, which were randomly chosen from green, blue, and red on each trial. One color was assigned to the target set, one to the distractor set. Yellow Gabor patches were always task-irrelevant and appeared on each search display; they could appear in any of the six orientations (please see the next section for details). Gabor patches with non-yellow colors were task-relevant; only one of them had the target orientation that was tilted to the left (− 45° for the easy task, or − 15° for the difficult task) or right (+ 45° for the easy task, or + 15° for the difficult task), whereas the remaining non-yellow Gabors were oriented at either 0° or 90° to prevent the target orientation from popping out (see Fig. 1A). All Gabor patches were placed on two imaginary, concentric square-shaped arrays centered on the central fixation, at the eccentricities of 3.23° (inner square) and 5.64° (outer square) of visual angles. These Gabor patches appeared along the vertical midline (two at the top, two at the bottom) and on two sides (four on the left, four on the right), with lateral items positioned 1.21° of visual angle above and below the horizontal midline. Gabor patches sharing the same color were spatially grouped, and placed either vertically (see Fig. 1C), or laterally to facilitate the implementation of future ERP studies.

Prior to each search display, a cue was presented. The cue consisted of a task-relevant colored circle (radius: 0.69°) at the center of the screen (see Fig. 1B). This circle contained a positive (+), negative (−), or neutral (○) symbol at its center. When a positive (+) symbol was shown, the color of the circle indicated the target color in the subsequent search display; when a negative (−) symbol was shown, the color of the circle indicated the to-be-ignored distractor color; when a neutral (○) symbol was shown, the circle’s color would not appear in the subsequent search display. The three task-relevant colors (red, green, blue) appeared with equal probability in all cue types with the restriction that the cued color would not repeat in successive trials.

### Procedure and design

As depicted in Fig. 1C, each trial started with a cue display that was presented for 250 ms. After a short delay of 900 ms, a search display appeared and stayed visible until a response was made. Participants were asked to search through eight non-yellow Gabor patches to find the target with a tilted orientation; participants reported whether it was tilted to the left or right. Responses were collected via a gamepad with two buttons for indicating left (←) and right (→) responses. Participants used their index and middle finger of the right hand to press the two buttons. They were instructed to respond as quickly and accurately as possible. In case of an erroneous or a slow response (> 4,000 ms), a white text with “incorrect” or “too slow” would appear for 500 ms on the screen. Each trial was followed by a blank interval for 1,000 ms. The three cue types were presented in separate blocks; the order followed a Latin-square design and was counterbalanced across participants. Participants were informed of the upcoming cue type before each block. Participants could take a small pause after every 30 trials. They also got feedback about mean accuracy and averaged response times after each block (60 trials).

Blocks could either consist of 80% search trials with an easy target (tilted ± 45° relative to vertical midline) or a difficult target (tilted ± 15° relative to vertical midline). In expected easy blocks, 80% of the trials contained the easy search target and 20% of the trials a difficult search target; this logic was reversed in expected difficult blocks. These two kinds of blocks were performed in separate sessions of nine blocks (three blocks per cue type). Each participant performed both sessions separated by a break of at least 5 min. Prior to the start of a new session, participants were informed of the easy to difficult search ratio and practiced 30 trials for each cue type. The session order was counterbalanced across participants.

### Data analysis

As a first step and to follow the conventional approach (see Zhang & Carlisle, 2023), we conducted a three-way repeated-measures ANOVA on mean values of response times (RTs). Error rates (M = 2.75%, SD = 0.16) were also recorded and analyzed. Since there was no accuracy–speed trade-off, we report error rates in supplementary Table 1 without further discussion. For the RT analysis, trials with response times faster than 200 ms or slower than 4,000 ms were discarded. Incorrect trials and trials in which the response times exceeded 2.5 SDs from the mean RT calculated separately for each condition were removed from the data. When the task difficulty matched the expected effort (i.e., easy target in easy blocks, or difficult target in difficult blocks), we labeled the trials as “expected”; when there was an expectancy mismatch (i.e., easy target in difficult blocks, or difficult target in easy blocks), we labeled the trials as “unexpected.” ANOVA factors included cue type (positive, negative, neutral), task difficulty (easy, difficult), and expectation (expected, unexpected). Hypothesis-driven custom contrasts were used to directly examine the impact of expected or actual effort on negative cueing effects (RT difference for neutral minus negative cues). The ANOVA was performed in JASP 0.18.3 (JASP Team, 2024). Effect sizes are reported in terms of partial η squared (η_p_^2^) for F tests. All p-values were Greenhouse–Geisser corrected for violations of sphericity. Bayes factors were obtained to further quantify the strength of evidence for or against the null hypothesis.

For a more fine-grained analysis we also applied a Bayesian multi-level model which was implemented via the Bambi package (Capretto et al., 2022), a frontend for implementing generalized linear mixed models and fitting them with PyMC (Abril-Pla et al., 2023). This approach enables hierarchical modeling of the response distributions, taking into account their variability within and between participants; uncertainty is preserved over all levels and reflected in the posterior estimates. In addition, this approach takes the fact into account that the population of the cells is not fully balanced in our design, a necessity when introducing rarer, unexpected events. To make meaningful estimates, we excluded trials with response times faster than 200 ms. In this model, we inverse-transformed the RTs (RS = 1/RT, see, e.g., Whelan, 2008). This quantity has a far less skewed distribution compared to raw RTs and is better represented by a normal distribution. Note that fitting log-normal or exGaussian likelihoods to RT distributions might even better represent their shape, however, in our tests we could not obtain stable samples with these distributions in complex hierarchical models. We used the Bambi package’s default priors, leading to the following model specification:

$$RS \sim Cue * Difficulty * Expectation + \left(Cue * Difficulty * Expectation \left| Participant\right.\right)$$

Priors:
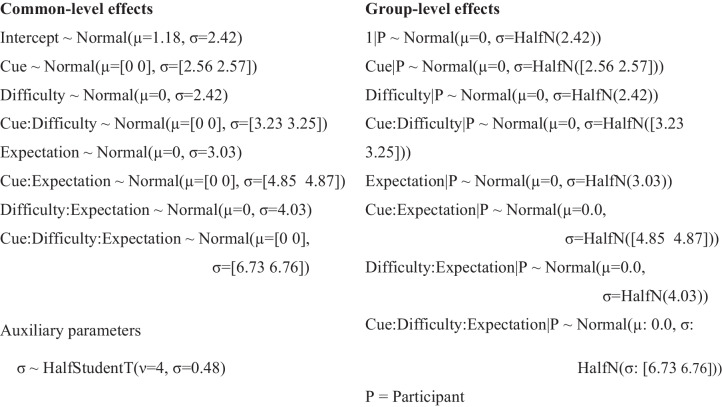


We sampled for 8,000 iterations in four chains using the NUTS sampler (Hoffman & Gelman, 2014). Convergence of the MCMC chains was confirmed visually and via the R̂ score (all R̂ = 1.0).

To interpret the outcomes of this model, we employed a marginal prediction approach (cf. Hanne et al., 2024) that transforms the effects back to the response scale, including undoing the inverse transformation, so that all outcomes are RTs in seconds. This process takes all the uncertainty information from the different model levels into account. On these quantities we calculated the contrasts of interest, reporting means and 95% highest-density intervals (HDI^95%^).

## Results

### ANOVA results

**Fig. 2:**
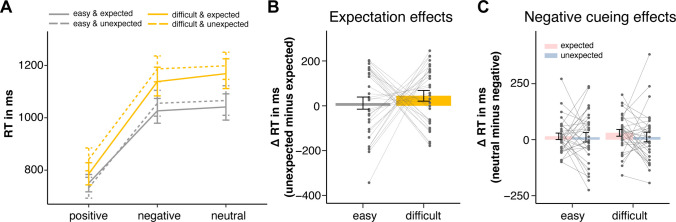
Response time (RT) results. (**A**) Mean RTs for expected and unexpected difficulty combinations as a function of cue type. The x-axis denotes the cue condition (positive, negative, and neutral cue); solid and dashed lines indicate whether the actual task difficulty matched participants' expectations (solid = expected; dashed = unexpected). Gray lines represent easy trials, and orange lines represent difficult trials. (**B**) Expectation effects (computed as RT difference of unexpected minus expected trials) in easy (gray) and difficult (yellow) search. (**C**) Negative cueing effects (computed as RT difference of neutral minus negative cue trials) for easy vs. difficult search, separated by whether the actual task difficulty matched participants’ expectation (pink) or did not match (blue). Error bars indicate standard error of means. Dots indicate individual data

We first report the main effects and interaction between cue type and task difficulty. As expected (see Fig. 2A), participants responded faster in easy search trials (M = 945 ms, CI^95%^ = [854, 1,036]) compared to difficult search trials (M = 1,053 ms, CI^95%^ = [962, 1,144]), F(1,29) = 105.87, p <.001, η_p_^2^ = 0.79, confirming the effectiveness of our difficulty manipulation. The main effect of cue type was also significant, F(1.44, 41.87) = 212.11, p <.001, η_p_^2^ = 0.89. Planned contrasts showed faster responses for positive cues (M = 778 ms, CI^95%^ = [685, 870]) than for neutral cues (M = 1,119 ms, CI^95%^ = [1,026, 1,211]), t(58) =  −18.27, p <.001, but response times were not significantly faster with negative (M = 1,102 ms, CI^95%^ = [1,009, 1,194]) compared to neutral cues, t(58) =  −0.90, p =.37.

The interaction between cue type and task difficulty was significant, F(2,58) = 10.08, p <.001, η_p_^2^ = 0.26; it was mainly driven by positive cueing effects (RT differences neutral minus positive cues) being smaller in easy search (M_diff_ = 312 ms, CI^95%^ = [264, 360]) compared to difficult search (M_diff_ = 370 ms, CI^95%^ = [322, 418]), F(1,29) = 20.39, p <.001, η_p_^2^ = 0.41. In contrast, negative cueing effects (RT differences neutral minus negative cues) did not differ between easy (M_diff_ = 13 ms, CI^95%^ = [−15, 40]) and difficult search (M_diff_ = 21 ms, CI^95%^ = [−7, 49]), F(1,29) = 0.37, p =.550, η_p_^2^ = 0.01.

Turning to whether participants’ expectations about task difficulty influenced RT performance, a main effect of expectation was observed: participants responded faster when task difficulty matched their expectation (M = 985 ms, CI^95%^ = [894, 1,076]) compared to when it did not (M = 1013 ms, CI^95%^ = [923, 1,104]), F(1,29) = 18.72, p <.001, η_p_^2^ = 0.39, indicating a general cost associated with effort mismatches (see Fig. 2B for details). However, neither the cue type × expectation interaction, F(2,58) = 0.99, p =.379, η_p_^2^ = 0.03, nor the task difficulty × expectation interaction, F(1,29) = 0.44, p =.515, η_p_^2^ = 0.02, nor the three-way interaction, F(2,58) = 1.21, p =.307, η_p_^2^ = 0.04, reached significance.

To directly assess the role of expected effort in negative cueing (see Fig. 2C), we contrasted negative cueing effects (RT differences neutral minus negative cues) in expected difficult trials with unexpected difficult trials. Results showed numerically larger cueing effects in the former (expected difficult: M_diff_ = 30 ms, CI^95%^ = [−16, 77]) compared to the latter (unexpected difficult: M_diff_ = 12 ms, CI^95%^ = [−35, 59]), but the difference was not significant, (expected minus unexpected: t(97.21) = 0.69, one-tailed p =.248), BF_10_ = 0.40, indicating that expecting a difficult trial does not foster the negative cue use. Similarly, no reliable difference in negative cueing effects was observed between expected easy (expected easy: M_diff_ = 15 ms, CI^95%^ = [−32, 62]) and expected difficult (expected difficult: M_diff_ = 30 ms, CI^95%^ = [−16, 77]) trials, (easy minus difficult: t(95.83) =  −0.58, one-tailed p =.281, BF_10_ = 0.46). Thus, also when comparing expected trials with differing task difficulty levels, the use of a negative cue was not more likely in more effortful searches.

### Bayesian multi-level model results

The Bayesian multi-level model largely confirmed the outcomes of the ANOVA, while also revealing additional insights. Material for conducting this analysis is available on our OSF repository (https://osf.io/bukpj/). Here, we focus on verifying negative cueing effects, their potential interaction with expectation and the role of effort expectation.

**Fig. 3:**
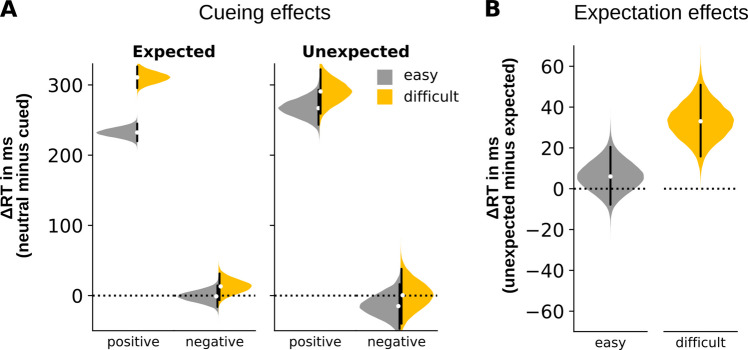
Posterior predictions of marginal effects from the Bayesian multilevel model. (**A**) Cueing effects (response time (RT) differences for “neutral” minus “positive” or “negative” cues) across task difficulty (easy vs. difficult) and expectation (expected vs. unexpected). Distributions in this figure are predicted via the Bayesian multi-level model and do not show the distributions raw data means (as in Fig. 2). Consequently, due to priors, shrinkage and other properties of the Bayesian multi-level model they can differ from the raw data means (as in Fig. 2). (**B**) Expectation effects (RT differences for “unexpected” minus “expected”), collapsed across cue types. The black vertical lines indicate the 95%-highest-density interval. The white dots reflect the distributions mode

As shown in Fig. 3A, negative cuing effects (RT differences neutral minus negative cues) were close to zero across all conditions. There was no meaningful difference of negative cueing effects between expected and unexpected difficult trials (expected minus unexpected: 13 ms, [−32, 57] HDI^95%^), confirming that expected effort did not modulate potential negative cueing effects. Furthermore, consistent with the earlier analyses, there was no reliable difference in negative cueing effects between expected easy and difficult trials (easy minus difficult: −14 ms, [−38, 11] HDI^95%^), further confirming that negative cueing effects were not modulated by actual effort.

Interestingly, the Bayesian model identified larger positive cueing effects (RT differences neutral minus positive cues) on unexpected compared to expected easy trials (unexpected minus expected: 35 ms, [7, 64] HDI^95%^, an interaction that was missed by the ANOVA. Further analysis revealed that this reduction in the cueing effects for easy trials was mainly driven by the positive cue: participants responded even faster when the easy trial was unexpected compared to when it was expected (unexpected minus expected: −22 ms, [−35, −9] HDI^95%^), suggesting that participants derive greater RT facilitation from positive cues in easy trials when they are expecting a difficult search.

In addition, although the general RT delays caused by expectation mismatch (collapsed across cue types) were replicated (see also Fig. 2B), the Bayesian model uncovered a task difficulty × expectation interaction (Fig. 3B) that was not detected in the ANOVA: A mismatch of expected and actual task difficulty resulted in RT delays in both trial types, yet this delay was estimated much smaller in easy trials (6 ms, [−8, 21] HDI^95%^) compared to difficult trials (33 ms, [16, 51] HDI^95%^; easy minus difficult: −27 ms, [−49, −4] HDI^95%^).

## Discussion

Although prior work showed evidence that negative cues are more effective under high search effort (Conci et al., 2019; Zhang et al., 2022), these studies did not clearly differentiate between actual and expected effort. There have also been reports of large individual differences in negative cue use (Chidharom & Carlisle, 2024; Heuer & Schubö, 2020), which might be caused by different expectations about cueing benefits and cue use effort. Therefore, we proposed that there might be a trade-off between cue use effort and the search effort participants expect in an upcoming task. Crucially, search effort needs to be predictable, so that the visual system can gauge their profitability sufficiently early. Following this, we assumed that the sometimes-elusive negative cueing effects could be strengthened in trials where participants expected a difficult search task compared to trials where an easy search was expected. To this end, we manipulated expectations by randomly embedding few easy target trials in blocks with mostly difficult target trials, or vice versa. Participants were clearly informed of upcoming easy-to-difficult ratios to foster stable expectations.

As assumed, participants responded faster during easy compared to difficult trials, confirming that the manipulation of target–distractor similarity successfully varied search difficulty and likely the experienced effort. Moreover, when the actual search difficulty in a trial matched the difficulty that was expected in the block, responses were overall faster than when it did not match. Such an expectation mismatch was reflected in an RT delay that was more pronounced in difficult compared to easy trials (Fig. 3B). Furthermore, positive cues yielded robust RT benefits, with larger cueing effects under high search difficulty, replicating prior findings that participants rely more heavily on positive cues when target–distractor similarity is high (Conci et al., 2019; Zhang et al., 2022). Zhang et al. (2022) using eye tracking found that participants made more target-directed fixations in difficult compared to easy trials, suggesting that positive cues help guiding attention more effectively under greater search difficulty. Also, they observed a correlation between fixation-based and RT benefits only in difficult trials, indicating that participants adaptively recruit cue information in response to more effortful search.

While some studies have also found larger negative cueing effects under high search effort (e.g., Arita et al., 2012; Conci et al., 2019; Zhang et al., 2022), our results did not show evidence for such an effect or its modulation by actual or expected effort. We consider it unlikely that we simply missed a substantial negative cueing effect due to insufficient statistical power, as our study was powered similarly to other studies that reported successful negative cueing. Furthermore, Bayes factors from the ANOVA contrasts were below 0.5. Given equal prior odds, this indicates that the null hypothesis was at least twice as likely as the alternative, providing weak evidence in favor of the null. As shown in Fig. 3A (left panel), marginal predictions for negative cueing effects in the expected difficult condition suggest a rough upper bound of about 32 ms (based on the upper HDI^95%^ boundary of the distribution shown in Fig. 3A, “expected”, “difficult”). This is about ten times lower than the positive cueing effect we observed, and smaller than the negative cueing effects typically reported in the literature (which are, e.g., ranging from 99 to 144 ms in the study by Arita et al. (2012); 206 ms in the study by Conci et al. (2019)). This suggests that the absence of a reliable negative cueing effect – and of any modulation of such an effect – in our data is more likely due to participants not, or only rarely, using negative cues, rather than merely reflecting an issue of experimental power.

To investigate whether design-related explanations can account for the lack of negative cueing effects in our study, we considered several key methodological factors: First, our cue was presented briefly (250 ms), which aligns with the durations used in Conci et al. (2019) and Zhang et al. (2022). Additionally, the cue-to-search display interval (900 ms) falls within the range of previously effective intervals – between 400–600 ms (Zhang et al., 2022) and 1,500–2,000 ms (Conci et al., 2019). Thus, it is unlikely that either insufficient cue exposure or temporal delay prevented participants from forming effective distractor templates. Second, in our task, the number of cued color items was four, that is, participants had to either search through four relevant items to identify the target, or they could ignore four non-relevant items (out of eight) in the search display. In previous studies that reported significant negative cueing effects (e.g., Conci et al., 2019; Zhang et al., 2022), participants could benefit from ignoring half of the display items – typically reducing their search size from eight to four items. This suggests that the proportional reduction in search size is comparable across studies. Therefore, the absence of an effect in our study is unlikely to result from differences in cueing efficiency. Third, we used block-wise manipulation of cue type (positive, negative, neutral), consistent with established protocols that promote stable cueing strategies (Arita et al., 2012; Conci et al., 2019; Zhang et al., 2022). One notable difference from prior studies is our use of spatially clustered displays, rather than random circular arrangements (e.g., Conci et al., 2019; Zhang et al., 2022). This design was motivated by broader research goals involving EEG measures, as separating target and distractor clusters spatially facilitates the dissociation of their neural signatures in ERP analyses. While differences in spatial clustering might introduce some variability in response times, we consider this as unlikely to explain the null result of negative cueing effects, because negative cueing effects did not differ between trials where the target appeared at vertical locations (M_diff_ = 17 ms, CI^95%^ = [−15, 49]) or at horizontal locations (M_diff_ = 17 ms, CI^95%^ = [−15, 49]), F(1,29) < 0.001, p =.974, and none of the interactions (from a repeated ANOVA with target location, task difficulty and expectation as factors) were significant (all ps >.050), suggesting that clustering plays a limited role. Supporting this, a prior study using a similarly clustered layout, also with a consistent task-irrelevant color, found the effectiveness of negative cues (Kerzel & Huynh Cong, 2022). These findings altogether suggest that display format alone cannot explain the null effects observed in the present study.

A more plausible explanation lies in the source of task difficulty. In the current study, task difficulty was increased by reducing target discriminability (Fig. 1 A), which primarily taxed perceptual processing. In contrast, prior studies reporting robust negative cueing effects often increased distractor interference, for example, by making distractors more similar to the target or increasing distractor set size (e.g., Arita et al., 2012; Kerzel & Huynh Cong, 2022; Zhang et al., 2022). This distinction may matter: when distractor interference is high, the need to overcome distractors increases, making negative cue use more advantageous. In such cases, participants may be more likely to proactively ignore or even suppress the distracting information, for instance, items of the cued, non-relevant color. That is, they may actively direct attention away from cue-matching distractor features before these items capture attention (Gaspelin et al., 2023; Liesefeld et al., 2024). This process typically involves effortful, proactive control to prevent interference (Chidharom & Carlisle, 2023; de Vries et al., 2019). In contrast, tasks that challenge perceptual discrimination of the target might not benefit from negative cue use in a similar way, making it unlikely that participants engage in effortful, proactive control. Our findings thus suggest that not all forms of increased task difficulty promote negative cue use in the same way.

This interpretation is consistent with neural findings that negative cue use depends on the proactive control-related brain mechanisms. Negative cues have been shown to elicit stronger midfrontal theta activity than positive and neutral cues, a neural marker associated with increased cognitive control engagement, suggesting that preparing a distractor template imposes mental effort (Chidharom & Carlisle, 2023; de Vries et al., 2019). This effort appears to be greater when negative cues change from trial to trial rather than remaining constant, reflecting additional effort associated with task-set switching (Cooper et al., 2019; Wen et al., 2022). Importantly, prior work has shown that the use of cognitively costly strategies depends not only on actual effort, but also on internal effort − reward trade-offs (Chong et al., 2017; Shenhav et al., 2017). Such a process is known to involve a distributed network of brain regions including the dorsomedial prefrontal cortex (dmPFC), ventromedial prefrontal cortex (vmPFC), and anterior cingulate cortex (ACC), which collectively evaluate whether deploying cognitive control is worthwhile (Aridan et al., 2019; Lopez-Gamundi et al., 2021; Massar et al., 2015; Prévost et al., 2010; Silvestrini et al., 2023; Vassena et al., 2014; Yao et al., 2023). In our study, even though participants expected high search effort, the nature of the task may not have justified the mental effort of using negative cues, providing little incentive for engaging proactive control mechanisms. As a result, these neural systems may have computed a low utility signal for using the negative cue, leading participants to disregard negative cues altogether.

Therefore, negative cueing effects may depend on the specific forms of search effort involved, highlighting the need to move beyond a unitary concept of “task difficulty” toward a more nuanced view of considering its cognitive composition. Neural mechanisms supporting distractor suppression – such as midfrontal regions – may be selectively recruited only when search tasks call for control-based efforts (e.g., Kerzel & Huynh Cong, 2022), rather than when perceptual demands dominate due to reduced target discriminability. Future research should aim to disentangle these different forms of search effort, both behaviorally and neurally, to clarify the boundary conditions under which negative cueing is effective.

Our results also revealed faster responses when task difficulty matched expectations compared to when it did not, a finding that aligns with earlier results showing that expectations enhance processing efficiency for high-probability stimuli (Jiang et al., 2016; Kok et al., 2012). This finding is consistent with predictive coding models (Huang & Rao, 2011; Rao & Ballard, 1999; Walsh et al., 2020; see also Slagter & van Moorselaar, 2021), which claim that the brain continuously generates predictions about forthcoming sensory input or task demands. When actual search effort diverges from expectations – such as when a task presumed to be easy turns out to be difficult – a prediction error may arise. To resolve this mismatch, the brain updates its internal models to match reality, a process that is time-consuming and involves increased engagement of brain regions, such as the insula and the inferior frontal gyrus (IFG), which are central to prediction error computation (Ficco et al., 2021; Weilnhammer et al., 2017).

Extending the literature regarding mismatch-related RT delays, our Bayesian model results revealed an interaction between expectation and task difficulty that was not detected by conventional ANOVA, highlighting the added value of model-based approaches in uncovering subtle, context-dependent effects that may be obscured by condition-level averaging. Specifically, the mismatch between expected and actual task difficulty incurred substantial RT delays in difficult trials, but showed rather small effects in easy trials (Fig. 3B). This relatively smaller delay observed in easy trials was mainly driven by positive cues (see appendix Fig. 4): positive cues made participants respond faster in unexpected easy trials (embedded in difficult blocks) compared to expected easy trials (embedded in easy blocks). This pattern seemingly contradicts predictions derived from the traditional predictive coding framework, which would suggest that expected trials should typically lead to faster responses.

To explain this counterintuitive result, we propose two possible accounts. The first one assumes that in difficult blocks, where targets are more difficult to identify, participants set up a narrowly tuned orientation template (± 15°) to identify the target. When an easy target with a larger tilt (± 45°) appears, this precise template suffices for efficient target detection, which is also facilitated by the more salient target orientation without requiring additional mental effort. While this account can explain the RT facilitation observed in unexpected easy trials, it fails to explain why such a benefit occurs only in positive cue conditions. A second, complementary account additionally assumes that in difficult search, participants use the positive cue to proactively update their target template to facilitate the search for the target. Recall that in difficult search blocks, participants can also rely on the fine-grained orientation information (± 15°) to set up a sufficiently precise, or “good-enough” target template (Geng et al., 2017; Yu et al., 2023). Yet integrating the information about the target color, as indexed by the positive cue, additionally allows for more efficient attentional guidance toward target items, and, as a consequence, speeds up search. When an easy trial unexpectedly appears, participants can benefit from both, more efficient attention guidance triggered by the positive cue, and the more pronounced orientation contrast of the easy target. In this case, the classical RT delay associated with the expectation mismatch gets overridden by the stronger facilitation caused by a combination of more fine-grained top-down attentional control (due to the more precise target template) and a more salient target, triggering bottom-up control. In contrast, under negative or neutral cue conditions, the target color remains unknown until search display onset, preventing participants from proactively adjusting attentional control settings. While both accounts suggest increased mental efforts during difficult blocks, they differ in the type of effort involved. The first account emphasizes perceptual effort and does not require frequent updating of the target template, whereas the second involves trial-by-trial updating of the attentional control settings to set up a “good-enough” target template that integrates both color and orientation information.

To distinguish between these two mechanisms, we examined whether unexpected easy trials with positive cues elicited post-conflict adjustments, indicative of increased cognitive control on the subsequent conflict trial (Botvinick et al., 2001; Kerns et al., 2004). Specifically, we compared RTs on difficult trials that followed either an unexpected easy trial (M = 802 ms, CI^95%^ = [696, 908]) or an expected difficult trial (M = 781 ms, CI^95%^ = [675, 888]). We observed significant post-conflict slowing in the former case, t(29) = 2.27, one-tailed p = .016, BF_10_ = 3.45, suggesting expectation mismatch prompted recalibration of attentional control settings. Notably, this post-conflict adjustment was absent when participants were expecting an easy search, t(29) =  −0.19, one-tailed p =.426, BF_10_ = 0.17. This indicates that control-related adjustments are more strongly triggered when participants are expecting a difficult search.

Therefore, our results reveal an interesting asymmetry in how expectation mismatch affects performance in easy and difficult trials in combination with a positive cue. In unexpected easy trials, responses were facilitated likely due to the more fine-grained attentional control state in anticipation of a difficult search. In contrast, unexpected difficult trials led to substantial RT delays compared to expected difficult trials, presumably because participants had to abruptly shift from a low attentional control state established in an easy block to a high attentional control state required for processing the unexpected difficult trial. This adjustment of attentional control appears to incur additional mental effort. Despite this, the mismatch did not reduce the positive cueing effects (neutral minus positive cues) in difficult trials (Fig. 3A, right panel).

The directional asymmetry observed with positive cues may also be reflected in the temporal dynamics of expectation mismatch processing. A longstanding debate in the literature concerns whether such mismatches influence early sensory processing (e.g., Egner et al., 2010; Jiang et al., 2013; Kok et al., 2012, 2017), or instead operate primarily at later, post-perceptual stages (Bang & Rahnev, 2017; Berggren & Eimer, 2019; Grubert & Eimer, 2023; Rungratsameetaweemana et al., 2018). While this debate has typically focused on perceptual expectations, our findings suggest that the directionality of effort-related mismatch may help reconcile these opposing views. Specifically, the facilitation observed in unexpected easy trials likely reflects preparatory control processes initiated prior to search display onset, which will enhance early sensory processing upon stimulus presentation, whereas the delays seen in unexpected difficult trials appear to reflect later adjustment recruited after display onset to meet increased task difficulty. In this way, the direction of effort-related expectation mismatch may map onto distinct temporal stages of attentional processing, offering a potential view for integrating prior diverging findings.

To conclude, although neither expected nor actual effort amplified any potential negative cue use to a detectable level in our study, linking our results to the literature suggests the importance of distinguishing different forms of search effort in negative cue use. Furthermore, the Bayesian model revealed asymmetric expectation effects in positive cue conditions. This elucidates how the brain integrates both bottom-up processing and top-down attentional control in the context of expectation mismatch involving low or high search effort, suggesting a boundary condition for predictive coding models in attentional control settings.

## Electronic supplementary material

Below is the link to the electronic supplementary material.

Supplementary file1 (DOCX 22 kb)

## Figures and Tables

**Fig. 4:**
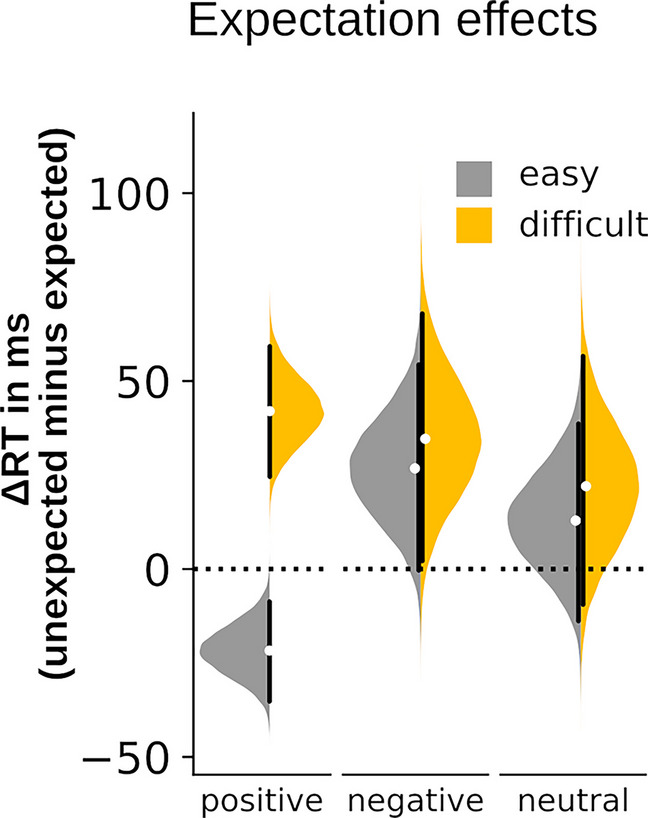
Expectation effects (RT differences for “unexpected” minus “expected”) from the Bayesian multilevel model for positive, negative and neutral cues separately. The black vertical lines indicate the 95%-highest-density interval. The white dots reflect the distributions mode

## Data Availability

Data for statistics reported I this work are available in the Open Science Framework (OSF) repository at https://osf.io/bukpj/.

## Code availability

The Bayesian model implementation is available in the Open Science Framework (OSF) repository at https://osf.io/bukpj/.

## Declarations

### Open practices statement

Other materials for the study, beyond those publicly shared, are available upon reasonable request. This study was not preregistered.
