# Supplementary material for: Effort expectation and strategic cue use in visual search
Source: Cogn Affect Behav Neurosci. 2025 Nov 11;26(2):505–17. doi: 10.3758/s13415-025-01358-1 (PMC13095900; doi:10.3758/s13415-025-01358-1)
Supplement: Supplementary file 1 — Supplementary file1 (DOCX 22 kb) [file 13415_2025_1358_MOESM1_ESM.docx]

**Supplementary Table 1.** Mean error rates (%) and its 95% confidence interval for the current study.

| Task difficulty | Cue type | Expectation | |
| --- | --- | --- | --- |
|  |  | expected | unexpected |
| easy | positive | 2.62 [1.40, 3.84] | 2.50 [1.28, 3.72] |
|  | negative | 3.43 [2.21, 4.65] | 3.52 [2.30, 4.74] |
|  | neutral | 3.52 [2.30, 4.74] | 3.43 [2.21, 4.65] |
| difficult | positive | 1.67 [0.45, 2.89] | 2.32 [1.10, 3.53] |
|  | negative | 2.43 [1.21, 3.65] | 2.59 [1.37, 3.81] |
|  | neutral | 2.66 [1.44, 3.88] | 2.96 [1.74, 4.18] |

Note. There was a main effect of cue type, F(2,58) = 3.65, p = .032, η_p_^2^ = 0.11. Planned contrasts showed that positive cues (M=2.27, CI^95%^ = [1.49, 3.06]) led to smaller error rates than neutral cues (M=3.14, CI^95%^ = [2.07, 4.21]), t(58) = -2.53, p =.014, while no difference was found between negative (M=2.99, CI^95%^ = [2.02, 3.97]) and neutral cues, t(58) = -0.44, p =.663. No other effects were significant (ps >.05).
